# Diagnostic Challenges of Non-Sporulating Fungal Isolates Recovered from Traumatic Wounds Following Motor Vehicle Collisions: *Subramaniula asteroides* and *Aspergillus quadrilineatus* in the United Arab Emirates

**DOI:** 10.3390/jof12090707

**Published:** 2026-09-21

**Authors:** Hari Pankaj Vanam, Deema Abdulrahim Alamoodi, Akela Ghazawi, Connie Cañete-Gibas, Nathan P. Wiederhold, Fatima Al Dhaheri

**Affiliations:** 1 Department of Pediatrics, College of Medicine and Health Sciences, Al Ain 15551, United Arab Emirates; 2 Department of Medical Microbiology and Immunology, College of Medicine and Health Sciences, Al Ain 15551, United Arab Emirates; 3 Fungus Testing Laboratory, Department of Pathology and Laboratory Medicine, University of Texas Health Science Center, San Antonio, TX 78229, USA; 4 Molecular Diagnostics Laboratory, Department of Pathology and Laboratory Medicine, University of Texas Health Science Center, San Antonio, TX 78229, USA

**Keywords:** *Subramaniula asteroides*, *Aspergillus quadrilineatus*, mycelia sterilia, non-sporulating

## Abstract

Environmental fungi may be recovered from traumatic wounds following motor vehicle collisions (MVCs), but distinguishing transient environmental inoculation, colonization, or contamination from true fungal infection can be challenging. Diagnostic challenges arise when fungal isolates recovered from clinical specimens remain non-sporulating (i.e., sterile), limiting conventional morphological identification and potentially remaining unidentified by routine laboratory platforms such as matrix-assisted laser desorption/ionization time-of-flight mass spectrometry (MALDI-TOF MS). We describe two cases involving the recovery and diagnostic characterization of *Subramaniula asteroides* and *Aspergillus quadrilineatus* from traumatic wounds following MVC-related polytrauma in the United Arab Emirates. In Case 1, a 55-year-old man with a severe crush–degloving hand injury underwent infectious disease evaluation for a suspected complicated skin and soft tissue infection (SSTI), prompting wound culture and antibacterial therapy. The recovered non-sporulating mold was identified as *S. asteroides* by ITS and D1/D2 sequencing and phylogenetic analysis; histopathologic assessment for fungal tissue invasion was not performed, no antifungal therapy was documented, and the wound improved following surgical and antibacterial management. In Case 2, a 40-year-old man with extensive upper-limb trauma underwent debridement and skin grafting, with wound culture yielding an initially non-sporulating mold subsequently identified as *A. quadrilineatus* by multilocus analysis of ITS, β-tubulin, and calmodulin. Voriconazole was initiated following communication of the species-level identification and discontinued after 15 days when the isolate was considered more likely to represent colonization or contamination than true wound infection. Although invasive fungal disease was not established in either case, these reports highlight the diagnostic challenges posed by non-sporulating molds, particularly in distinguishing fungal recovery from true infection in traumatic wounds. They also demonstrate the value of reference mycology laboratories integrating phenotypic and molecular approaches in accurately identifying uncommon environmental fungi and informing the interpretation of their clinical significance.

## 1. Introduction

Traumatic injuries involving extensive soft tissue disruption and environmental contamination may facilitate direct inoculation of environmental fungi into damaged tissue. Although post-traumatic invasive fungal infections (IFIs) are uncommon, they can cause substantial morbidity and mortality, particularly following severe polytrauma, crush injuries, and tissue devitalization [1,2,3]. Importantly, fungal recovery from a traumatic wound itself does not establish fungal infection or tissue invasion and should be interpreted in conjunction with clinical, microbiological, and, where available, histopathologic findings.

Among environmental molds, *Aspergillus* species are ubiquitous and cause a spectrum of diseases ranging from superficial and cutaneous manifestations to invasive aspergillosis [4,5]. Cutaneous aspergillosis is uncommon and may result from direct traumatic inoculation or disseminated disease, particularly in critically ill or immunocompromised patients [4,5,6]. Primary cutaneous aspergillosis has been associated with agricultural and orthopedic trauma, burns, surgery, and traffic-related injuries [4]. The principal species implicated include *A. fumigatus*, *A. flavus*, *A. niger*, *A. terreus*, and *A. ustus*, with *A. fumigatus* and *A. flavus* among the most frequently reported [7].

Less commonly encountered and cryptic *Aspergillus* species pose additional identification challenges [5,8]. *Aspergillus quadrilineatus*, a member of section *Nidulantes*, is infrequently reported from clinical specimens but has been associated with fungal sinusitis, onychomycosis, central nervous system infection, and pulmonary infection [9,10,11,12]. Recent epidemiologic mapping documents *A. quadrilineatus* among *Aspergillus* species reported from the Middle East [5], while a molecular epidemiological study from Qatar identified it in only 1 of 70 clinical *Aspergillus* isolates (1.4%) [13]. These observations document its occurrence within the Arabian Gulf while emphasizing its rarity among clinically recovered *Aspergillus* isolates.

*Subramaniula asteroides* is an infrequently reported *Chaetomium*-like environmental fungus whose prevalence among clinical isolates remains unknown and may be underestimated because of difficulties in phenotypic identification [14]. Clinical isolates have been reported predominantly in association with ocular disease, particularly keratitis, with skin and sinus involvement also described [14,15,16]. Environmental isolates have also been recovered from arid soils in Saudi Arabia and Egypt [14,17]. *Chaetomium*-like fungi have been associated with traumatic inoculation, and *S. asteroides* has been recovered from both clinical and environmental sources [14,15]. Identification may be challenging because these fungi can fail to develop diagnostic reproductive structures and consequently present as non-sporulating or “sterile” mycelia; molecular characterization is therefore important for resolving morphologically inconclusive isolates [14,15].

Non-sporulating molds recovered from clinical specimens present a broader diagnostic challenge because the absence of reproductive structures can preclude conventional morphological identification [14,18,19,20]. Matrix-assisted laser desorption/ionization time-of-flight mass spectrometry (MALDI-TOF MS) provides rapid identification but depends on adequate representation within reference spectral databases and may have limited discriminatory capacity for uncommon or closely related taxa, including members of *Aspergillus* section *Nidulantes* [5]. Molecular sequencing provides an important complementary approach; for *Aspergillus*, loci such as β-tubulin (*benA*) and calmodulin (*CaM*) can improve species-level resolution when used alongside the internal transcribed spacer (ITS) region [5,8,21]. Such isolates may therefore benefit from referral to specialized mycology laboratories with molecular identification capabilities. Accurate species-level identification is clinically relevant because uncommon and cryptic molds may differ in antifungal susceptibility, while established interpretive criteria for rare species may be limited or unavailable [5,15].

The objective of this study was to describe the clinical context and laboratory characterization of two uncommon fungal isolates, *S. asteroides* and *A. quadrilineatus*, recovered from traumatic wounds following motor vehicle collision (MVC)-related polytrauma in the United Arab Emirates, highlighting both the challenges of species-level identification and the clinical interpretation of unusual fungal recovery from traumatic wounds.

## 2. Case Reports

**Figure 1:**
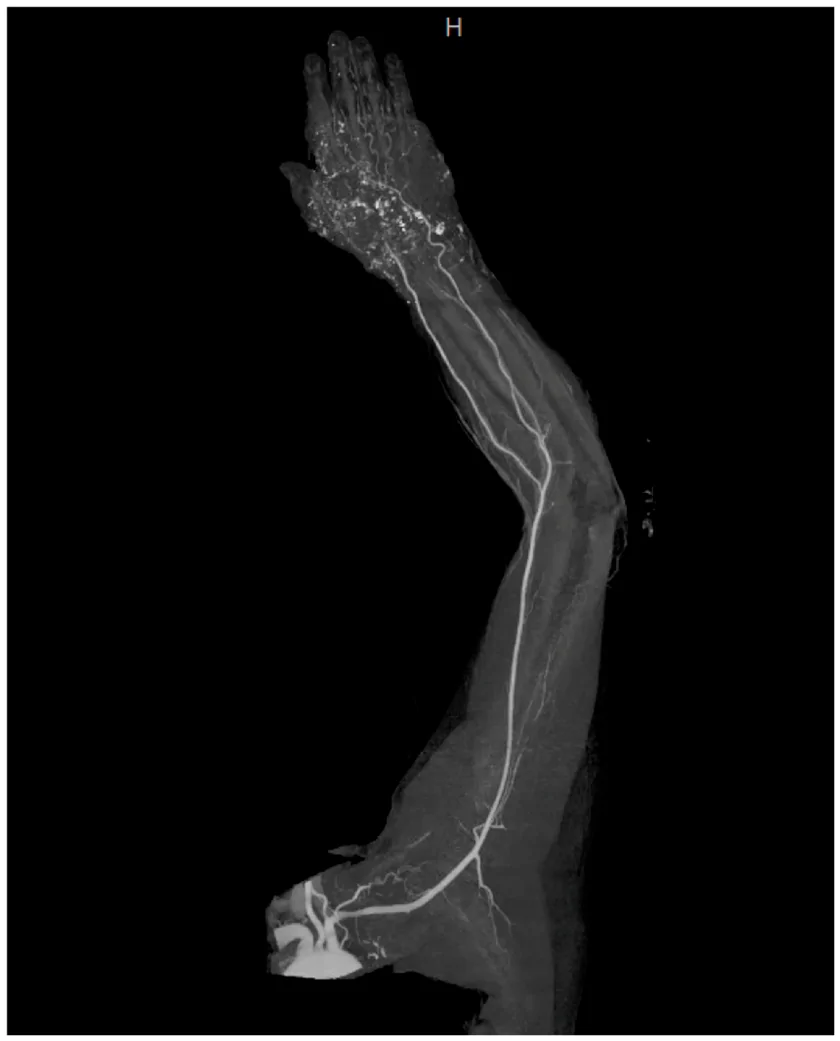
CT angiography of the left upper extremity following motor vehicle collision in Case 1. Three-dimensional angiographic reconstruction demonstrating distal radial artery injury at the level of the wrist. Associated CT findings included extensive carpal and carpometacarpal fracture–dislocations, including displaced comminuted fractures of the hamate and pisiform, dislocation of the trapezium and trapezoid, and fractures of the fourth and fifth metacarpal bases, with surrounding hematoma and surgical emphysema extending into the forearm.

Case 1: A 55-year-old Pakistani man with stage 5 chronic kidney disease (CKD) presented to the emergency department following a motor vehicle collision (MVC), sustaining a severe crush–degloving injury to his left hand with active bleeding (Day 0). Initial trauma assessment revealed fractures and dislocations involving the distal radius, ulnar styloid, carpal bones, fourth/fifth carpometacarpal joints, and proximal phalanx of the thumb, alongside distal radial artery injury and surgical emphysema. Computed tomography (CT) angiography demonstrated distal radial artery injury at the wrist, advanced carpal and carpometacarpal fracture–dislocation with multiple bony fragments and surrounding hematoma, distal radioulnar joint subluxation, and surgical emphysema extending into the forearm (Figure 1). Emergency management included single intravenous (IV) doses of cefazolin 1 g and gentamicin 80 mg, both renally adjusted, after which the patient was transitioned to amoxicillin–clavulanic acid 1 g every 8 h. Tetanus prophylaxis was also provided. Orthopedic and plastic surgery teams performed immediate debridement, hemorrhage control, temporary K-wire fixation of the first to third metacarpals to the radius because of extensive soft tissue loss, and wrist semi-amputation. Intraoperative findings revealed complete thumb avulsion and extensive destruction of the thenar, hypothenar, adductor, lumbrical, and interosseous muscle compartments.

Postoperatively, on Day 15, infectious disease evaluation documented concern regarding a postoperative complicated skin and soft tissue infection (SSTI) involving the traumatic wound, with the clinical assessment noting persistent leukocytosis (WBC 13.4 × 10^9^/L; reference range, 4.5–11.0 × 10^9^/L). The patient was afebrile, and dressing change revealed no bleeding or discharge. The flap was healthy and warm, with capillary refill of 2–3 s and a triphasic Doppler signal, and the donor site was dry. C-reactive protein was high at 17.7 mg/L while procalcitonin was normal (0.07 ng/mL). Piperacillin–tazobactam 4 g IV every 8 h and vancomycin 1.25 g IV every 12 h were initiated on Day 15 and continued for a total of 8 days. The patient was transferred to another acute care facility on Day 16. By Day 19, the wound was documented as clean and healing. Oral levofloxacin and linezolid were initiated on Day 22 with a plan to continue them for an additional 7 days; however, on Day 25, due to intolerability, antibiotics were discontinued, while the wound was described as clean and dry.

On Day 26, a specimen from the left wrist was collected for microbiological culture in the context of new hospital-acquired fever. Empiric antibacterial therapy with piperacillin–tazobactam 2 g IV every 8 h and linezolid 600 mg IV every 12 h was initiated. Direct examination of the original clinical specimen by Gram stain showed Gram-positive cocci (bacterial elements were not isolated); however, fungal elements were observed on Day 28. No clinical intervention by the treating team was documented following this result. Fungal growth was observed after 9 days (Day 36 after patient was discharged). Microscopic examination of the cultured isolate demonstrated heterogeneous hyphal morphology, including hyaline and darker thick-walled elements. The isolate was provisionally identified as *Scytalidium* sp. based on its cultured phenotypic features, including woolly gray colonies with a dark reverse and septate branching hyphae with arthroconidial elements. However, the absence of diagnostic sporulation precluded definitive phenotypic identification, and MALDI-TOF MS (VITEK MS; bioMérieux, Marcy-l’Étoile, France) did not provide a species-level identification. Definitive reconstructive surgery included wrist arthrodesis and a microsurgical free anterolateral thigh flap with split-thickness skin grafting. The patient was discharged on Day 32 and subsequently attended an outpatient wound management clinic for dressing follow-up on Day 35, with no clinical concerns documented.

The fungal isolate was referred to the UAE University Fungal Reference Laboratory (UAEU FRL) on Day 57 for detailed phenotypic and molecular characterization as part of passive surveillance. ITS and D1/D2 sequencing, together with phylogenetic analysis, supported species-level identification of *Subramaniula asteroides*. Histopathologic assessment for fungal tissue invasion was not performed, and no antifungal therapy was documented. The mold was recovered only once, and no specific clinical response to the finding or initiation of antifungal therapy was documented, suggesting that the isolate was not considered clinically significant at the time.

**Figure 2:**
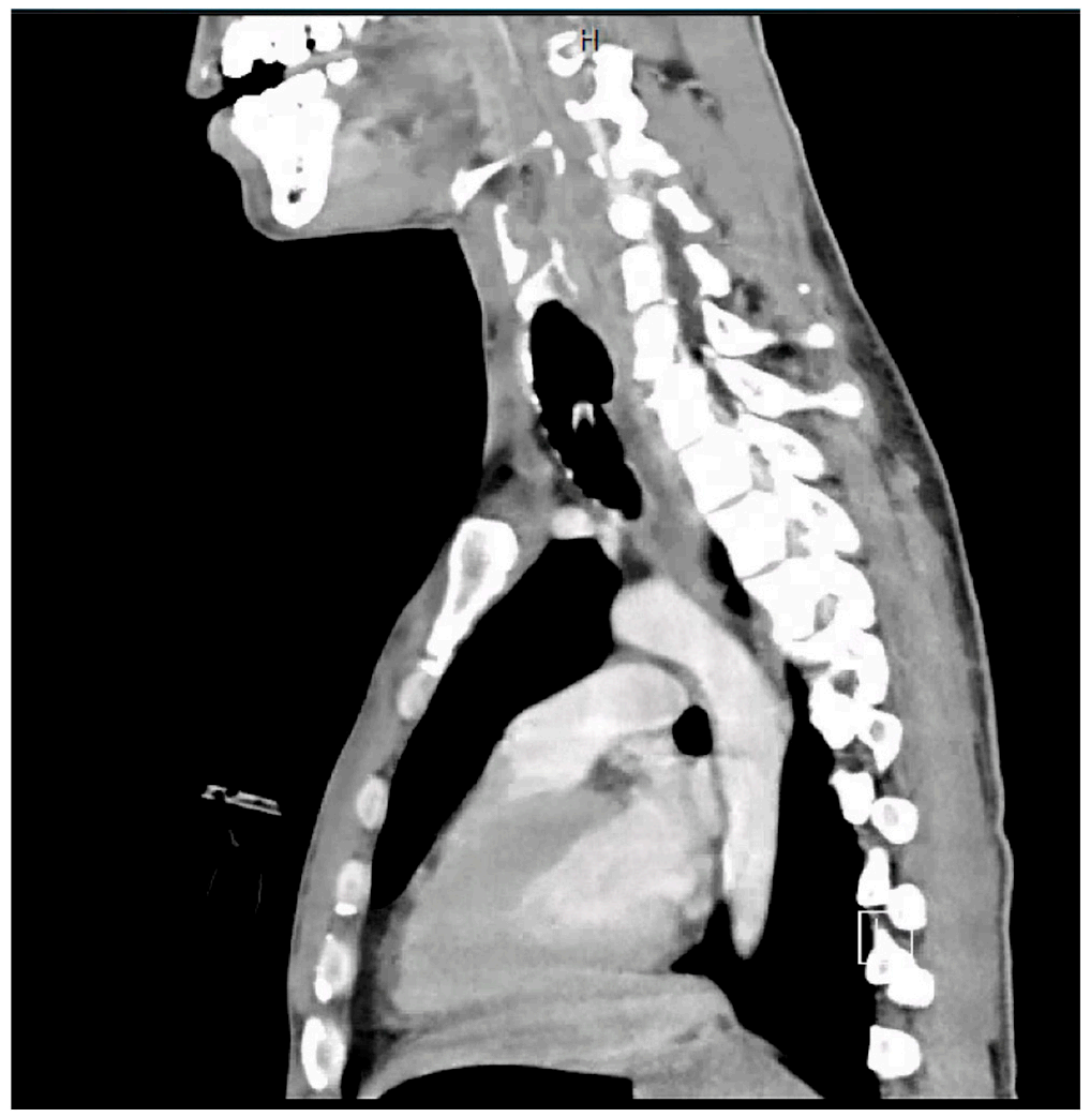
Contrast-enhanced sagittal CT image following the motor vehicle collision in Case 2, demonstrating extensive traumatic soft tissue injury involving the chest wall. The complete CT examination demonstrated a large right lateral and posterior chest wall hematoma extending into the right axillary region and toward the root of the neck.

Case 2: A 40-year-old Kenyan man with chronic kidney disease (Stage 4 CKD) was admitted to the intensive care unit (ICU) following polytrauma from a motor vehicle collision (MVC) (Day 0) and required mechanical ventilation. Renal function testing on admission revealed a serum creatinine of 106 µmol/L (micromoles per liter) and an estimated glomerular filtration rate (eGFR) of 74 mL/min. His injuries included an undisplaced fracture of the anterior arch of C1, a left occipital condyle fracture, a right C7 transverse-process fracture, a right first rib fracture, a small right-sided pneumothorax, multiple pulmonary contusions, a displaced right humeral shaft fracture, and complete cut-off of the right axillary artery at its origin. CT trauma imaging additionally demonstrated a large right lateral/posterior chest wall hematoma extending into the right axilla and root of the neck (Figure 2). On Day 1, he underwent emergent right subclavian–axillary artery grafting, right arm and forearm fasciotomies, brachial plexus repair, and external fixation of the right humerus.

Intravenous piperacillin–tazobactam 4 g every 8 h and vancomycin 1 g every 12 h were administered during the ICU admission. On Day 7, surgical debridement of devitalized tissue from the right arm and forearm fasciotomy wounds was performed, followed by partial primary closure and split-thickness skin grafting using a graft harvested from the right thigh. On the same day, a right arm wound specimen was collected and submitted for microbiological culture. Fungal elements were reported on Day 10, followed by a designation of “presumptive *Aspergillus*” on Day 20.

The cultured isolate was initially non-sporulating, precluding conventional morphological identification. MALDI-TOF MS (VITEK MS; bioMérieux) did not provide a species-level identification. The isolate was referred to the UAEU FRL on Day 18 for further characterization. Phenotypic examination and initial ITS sequencing placed the isolate within *Aspergillus* section *Nidulantes*. Subsequent *benA* and *CaM* sequencing, together with multilocus phylogenetic analysis, supported species-level identification as *Aspergillus quadrilineatus*. The identification was communicated to the clinical team on Day 30.

Voriconazole was initiated on Day 30, when the infectious disease team was notified, with a loading dose of 6 mg/kg every 12 h followed by a maintenance dose of 4 mg/kg every 12 h. On Day 45, On Day 45, voriconazole was discontinued after the isolate was considered more likely to represent contamination or colonization than true wound infection.

## 3. Materials and Methods

### Phenotypic and Molecular Characterization of Isolates

Primary microbiological processing of the clinical specimens was performed at the diagnostic (referral) laboratory using commercially prepared routine bacteriological and mycological culture media supplied by Medysinal FZCO (Dubai, UAE). For Case 1, the left-wrist specimen was cultured on mycological media, including Sabouraud dextrose agar (SDA), potato dextrose agar (PDA), and Mycosel agar, at 25 °C. Fungal growth was observed after 9 days of incubation, and the recovered mold was inhibited on the cycloheximide-containing selective medium. For Case 2, the right-arm wound specimen underwent routine bacteriological and mycological culture, including culture on PDA at 25 °C.

At the UAEU FRL, both recovered isolates were subcultured for phenotypic characterization on media prepared from commercially available dehydrated formulations supplied by HiMedia Laboratories Pvt. Ltd. (Mumbai, India), with the corresponding HiMedia product codes: Potato Dextrose Agar (PDA; M096), Malt Extract Agar Base with Mycological Peptone (MEA; M137), and Oatmeal Agar (OA; M397). PDA, MEA, and OA were prepared at the UAEU FRL according to the manufacturer’s instructions. Cultures were incubated at 28 °C, with parallel incubation at 42 °C to assess thermotolerance. Macroscopic characteristics were recorded during early growth, at maturity, and after prolonged incubation (>2 months). The features evaluated included colony texture, surface topography, radial folding, zonation, obverse and reverse pigmentation, margins, and exudate production. Microscopic examination was performed using slide cultures, adhesive tape mounts, and tease mounts prepared in either an in-house-prepared 10% aqueous lactic acid solution (10% LA) or commercially prepared Lactophenol Cotton Blue solution (LPCB; S016; HiMedia Laboratories Pvt. Ltd.).

For the Case 1 isolate, slide cultures were prepared on Water Agar (WA; HiMedia M1366) and Banana Leaf–PDA (BL-PDA), prepared using PDA (HiMedia M096) supplemented with sterilized banana leaf material to enhance the development of diagnostic microscopic structures. The media were prepared at the UAEU FRL according to the manufacturer’s instructions. Urease activity was assessed using commercially prepared urea agar slants (Cat. No. 432196; Medysinal FZCO) at 30 °C for 7 days. For the Case 2 isolate, early LPCB preparations from approximately 10-day-old cultures incubated at 28 °C beneath sterile cellophane facilitated detection of vesicle formation. Tease mounts and PDA slide cultures were subsequently examined after prolonged incubation at 28 °C for >2 months, when teleomorphic structures became evident.

For molecular characterization, genomic DNA was extracted using the Fungi/Yeast Genomic DNA Isolation Kit (Norgen Biotek Corp., Thorold, ON, Canada). Species-level identification was performed using multilocus sequencing. The internal transcribed spacer (ITS) region was amplified using primers BMB-CRF and ITS4-R [22] for preliminary taxonomic placement of the isolates. The *Aspergillus* isolate was further characterized by sequencing *benA* and *CaM* [23], whereas the Case 1 isolate was further characterized by sequencing the D1/D2 domains of the 28S rRNA gene [24].

Phylogenetic analysis was performed by aligning the generated sequences with reference sequences retrieved from GenBank in MEGA X v10.2.6 [25]. Maximum-likelihood trees were inferred in IQ-TREE v3.1.3 [26], with the best-fit nucleotide substitution model selected by ModelFinder according to the Bayesian information criterion (BIC). Branch support was assessed using 1000 ultrafast bootstrap replicates, 1000 SH-aLRT replicates, and the approximate Bayes test. Support values are reported as indicated in the corresponding figure legends. Trees were visualized using iTOL v7 [27].

## 4. Results

### 4.1. Phenotypic and Morphological Identification

**Figure 3:**
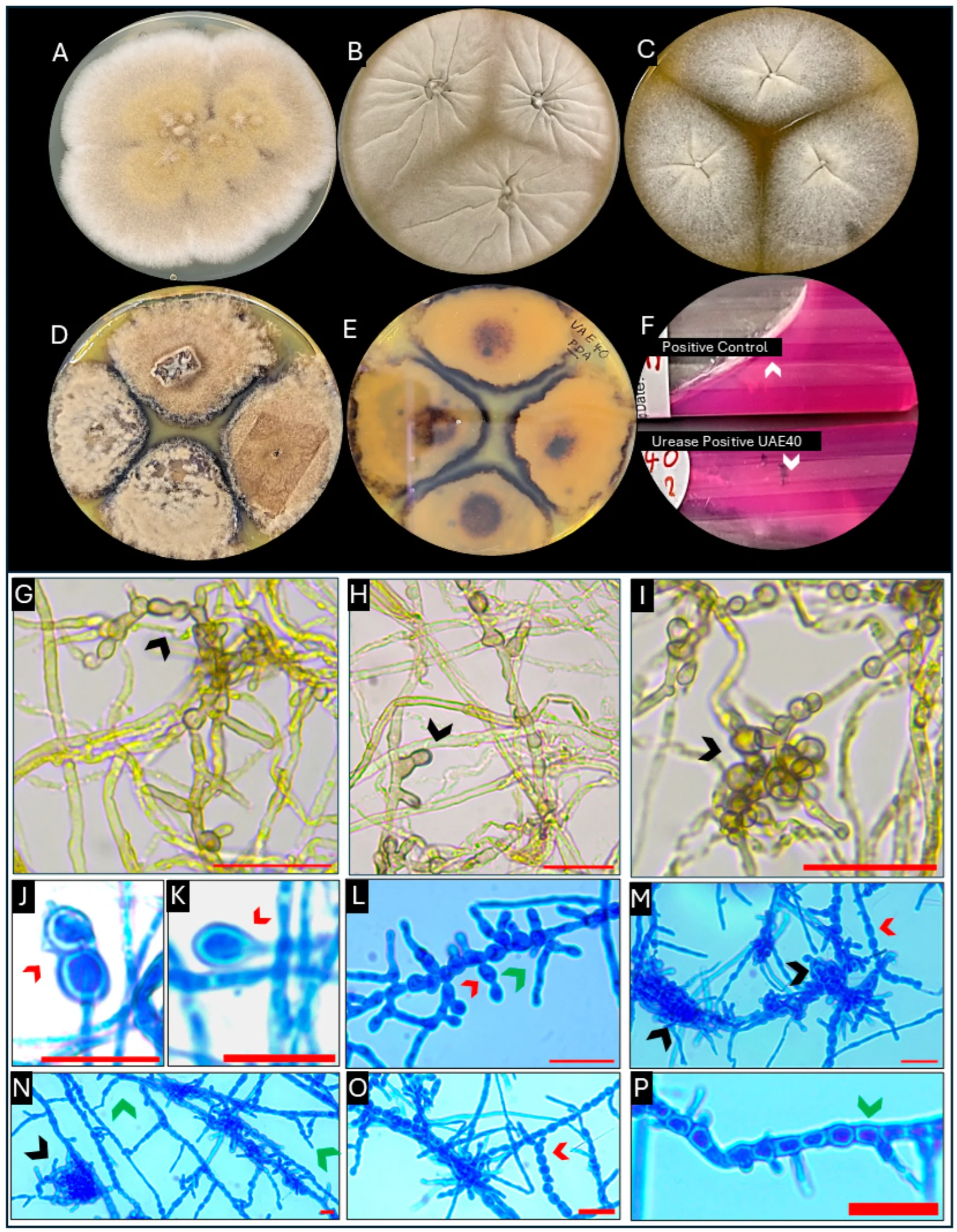
Phenotypic characteristics of *Subramaniula asteroides*. (**A–C**) Colony morphology on PDA at 28 °C (**A**), OA at 42 °C (**B**), and MEA at 42 °C (**C**). Growth on OA and MEA at 42 °C demonstrates thermotolerance. (**D**,**E**) PDA culture after prolonged incubation (>2 months), showing a thick, dry, crust-like colony with pronounced zonation on the obverse (**D**) and dark brown to black pigmentation on the reverse (**E**). (**F**) Positive urease reaction after 7 days of incubation at 30 °C (test isolate compared with positive control). (**G**–**I**) Further 10% lactic acid mounts demonstrating heterogeneous septate hyphae with intercalary papulospore-like, thick-walled elements and cellular aggregates (black arrowheads). (**J**–**P**) LPCB mounts demonstrating heterogeneous microscopic features, including short terminal or intercalary phialidic structures with sparse hyaline conidia (red arrowheads), compact bulbil-like aggregates composed of thick-walled cells (black arrowheads), and thick-walled septate hyphal/conidiogenous elements (green arrowheads). Scale bars = 20 µm.

Case 1 isolate: Colonies on PDA at 28 °C were white to pale yellowish-gray and floccose, with central olivaceous–black pigmentation (Figure 3A). Growth on OA and MEA at 42 °C demonstrated thermotolerance (Figure 3B,C). After prolonged incubation (>2 months) on PDA, colonies became thick, dry, and crust-like, with pronounced zonation and dark brown to black reverse pigmentation (Figure 3D,E). Urease activity was positive after 7 days of incubation at 30 °C (Figure 3F). Microscopic examination demonstrated heterogeneous septate hyphae, intercalary papulospore-like elements, phialidic conidiogenesis, and compact bulbil-like structures (Figure 3G–P).

**Figure 4:**
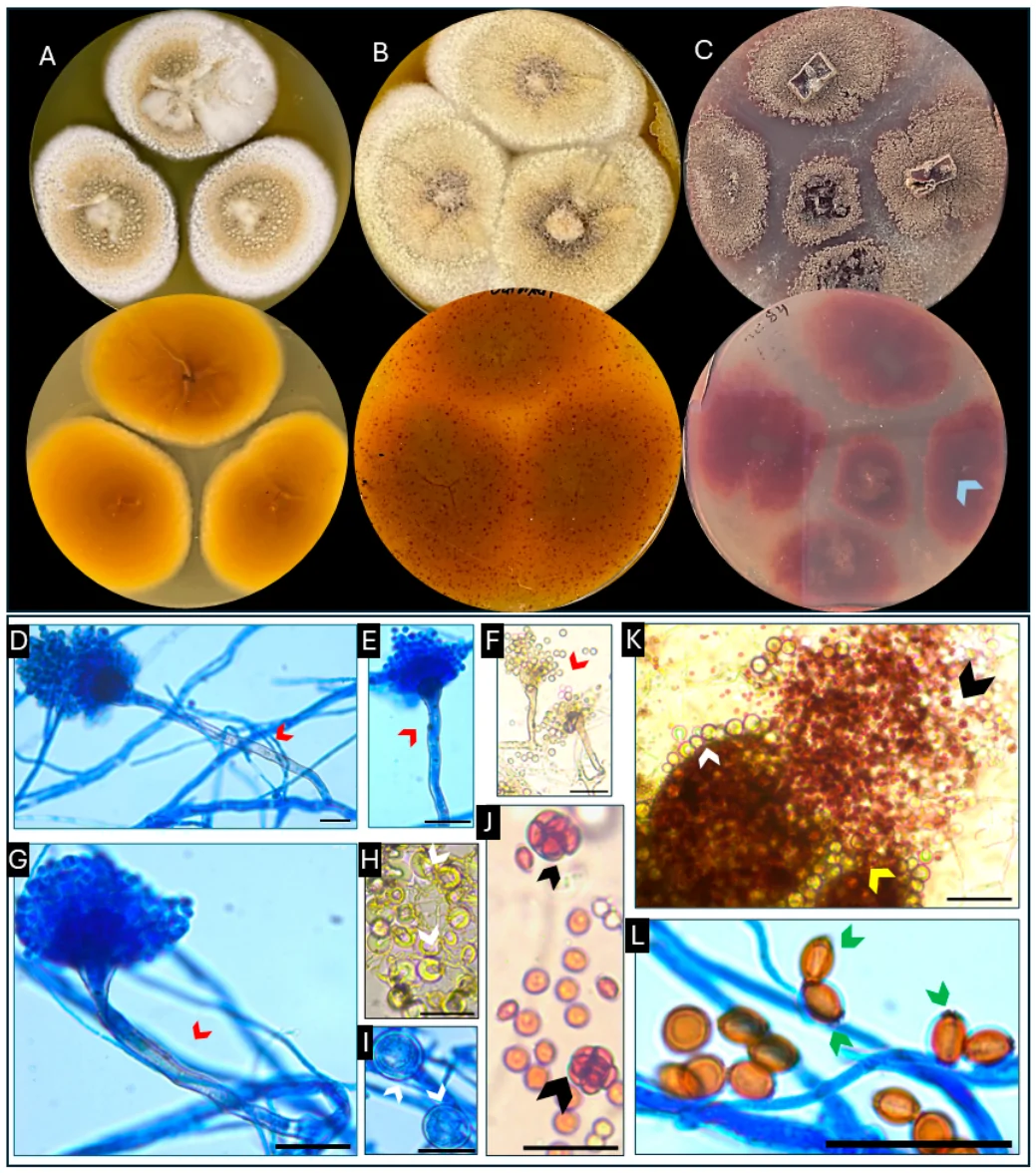
Phenotypic characteristics of *Aspergillus quadrilineatus*. (**A**–**C**) Colony morphology showing obverse and reverse views on MEA (**A**), OA (**B**), and PDA (**C**), illustrating progression from white floccose growth to sulcate, radially folded colonies with dark reddish-brown pigmentation and diffusible reddish-burgundy pigment after prolonged incubation at 28 °C (**C**, blue arrowhead). (**D**–**G**) Anamorphic structures showing short, smooth-walled brown conidiophores terminating in flask-shaped to hemispherical vesicles bearing biseriate conidiogenous cells (red arrowheads). (**H**,**I**) Hülle cells (white arrowheads). (**J**–**L**) Teleomorphic structures such as mature cleistothecium (**K**, yellow arrowhead), which released asci containing lenticular ascospores (**J**, black arrowhead) and mature lenticular ascospores with equatorial crests (**L**, green arrowheads). Microscopic preparations in panels (**F**,**H**,**J**,**K**) were mounted in 10% lactic acid; all other microscopic panels were prepared with LPCB. Scale bars = 20 µm.

Case 2 isolate: The isolate was initially non-sporulating, with expanding white colonies. Early microscopic examination of cultured material on PDA revealed developing *Aspergillus*-like vesicular structures with pigmented stipes, supporting placement within *Aspergillus* section *Nidulantes*. Colonies on MEA, OA, and PDA at 28 °C progressed from white floccose growth to sulcate, radially folded colonies with dark reddish-brown pigmentation and clear exudate droplets on the obverse; a diffusible reddish-burgundy pigment developed after prolonged incubation (Figure 4A–C). Microscopic examination demonstrated short, smooth-walled brown conidiophores terminating in flask-shaped to hemispherical vesicles with biseriate conidiogenous cells (Figure 4D–G), together with Hülle cells (Figure 4H,I). Following prolonged incubation, teleomorphic structures became evident, including mature cleistothecia, and they released asci containing lenticular ascospores and mature ascospores bearing equatorial crests (Figure 4J–L).

### 4.2. Molecular Identification

**Figure 5:**
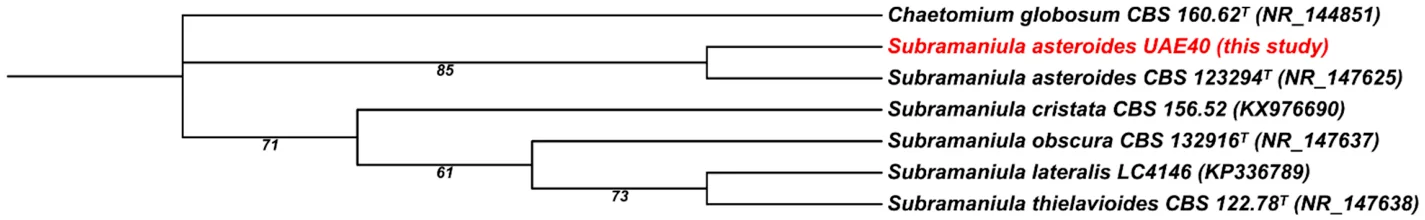
Maximum-likelihood phylogenetic tree of isolate UAE40 (shown in red) and reference strains of *Subramaniula* species, with *Chaetomium globosum* as outgroup, based on internal transcribed spacer (ITS) rDNA sequences. The tree was inferred using IQ-TREE v3.1.3 under the TN + F + I substitution model selected by ModelFinder according to the Bayesian information criterion (BIC). Branch support was assessed using 1000 ultrafast bootstrap (UFBoot) replicates, 1000 SH-aLRT replicates, and the approximate Bayes test; ultrafast bootstrap values (%) are shown at the nodes. Ex-type strains are indicated by a superscript T, and GenBank accession numbers are given in parentheses. *Chaetomium globosum* CBS 160.62ᵀ was used as the outgroup.

*Subramaniula asteroides* (Case 1): Molecular analysis of isolate UAE40 included sequencing of the ITS region and the D1/D2 domains of the 28S rRNA gene. For the ITS region, UAE40 showed 98.95% sequence identity (100% query coverage) with the ex-type strain of *S. asteroides* CBS 123294 (GenBank accession no. NR_147625). For the D1/D2 region, UAE40 showed 100% sequence identity (100% query coverage) with the ex-type strain of *S. asteroides* CBS 123294 (GenBank accession no. NG_246169). Maximum-likelihood analysis of the ITS region recovered UAE40 as sister to the ex-type strain of *S. asteroides* CBS 123294ᵀ (UFBoot = 85; Figure 5), within a monophyletic *Subramaniula* clade rooted with *Chaetomium globosum*. The sequences generated in this study were deposited in GenBank under accession numbers (UAE40-ITS: PZ274405 and UAE-40-D1-D2: PZ274423).

**Figure 6:**
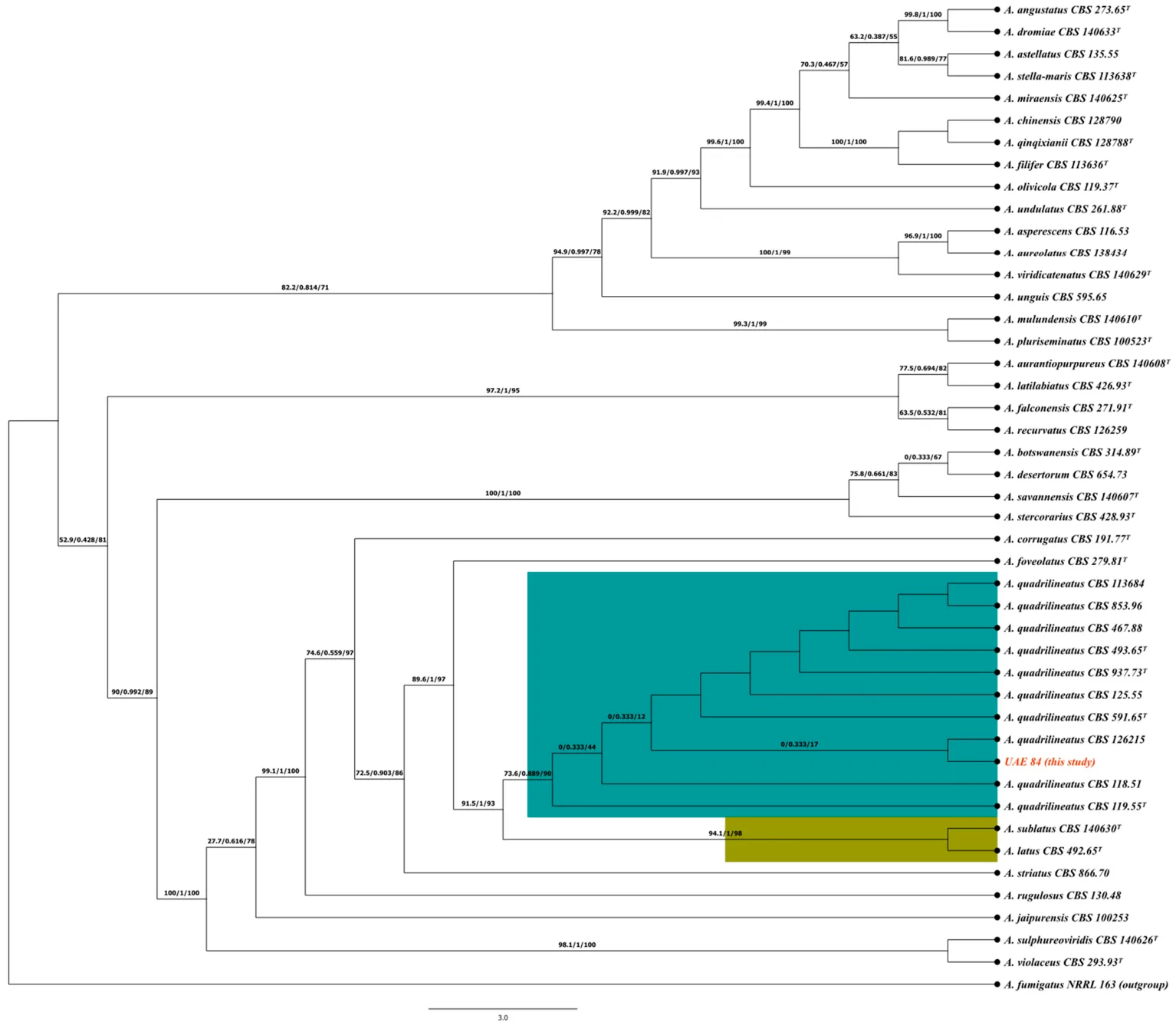
Maximum-likelihood tree showing isolate UAE84 clustered with *Aspergillus quadrilineatus*, based on a concatenated dataset of ITS, *benA*, and *CaM*. The tree was inferred using IQ-TREE v3.1.3 under the TIM + F + I + G4 substitution model selected by ModelFinder according to the Bayesian information criterion (BIC). Branch support was assessed using 1000 ultrafast bootstrap replicates, 1000 SH-aLRT replicates, and the approximate Bayes test, and is shown at the nodes as SH-aLRT (%)/aBayes/ultrafast bootstrap (%). The *A. quadrilineatus* clade is highlighted in teal, and the closely related sister species *A. sublatus* and *A. latus* are highlighted in olive. The isolate from this study (UAE 84) is shown in red. The tree was rooted with *Aspergillus fumigatus* NRRL 163. Ex-type strains are shown in bold and indicated by a superscript T.

*Aspergillus quadrilineatus* (Case 2): Multilocus phylogenetic analysis of isolate UAE84 using ITS, *benA*, and *CaM* supported species-level identification as *A. quadrilineatus* (Figure 6). Although ITS and benA did not discriminate UAE84 from the closely related *A. sublatus* and *A. nidulans* (ITS 99.56–99.78%; *benA* 99.13–100%), *CaM* provided greater discrimination of *A. quadrilineatus* (98.89%) from *A. sublatus* (97.33%) and *A. nidulans* (94.67%). Concatenated analysis of the three loci placed UAE84 within the *A. quadrilineatus* clade, alongside the ex-type strain CBS 591.65ᵀ, and resolved this clade as distinct from the sister clade comprising *A. sublatus* (CBS 140630ᵀ) and *A. latus* (CBS 492.65ᵀ; 94.1/1/98). UAE84 grouped most closely with *A. quadrilineatus* CBS 118.51 and CBS 119.55ᵀ (73.6/0.889/90). The sequences generated in this study were deposited in GenBank under accession numbers (UAE84-ITS: PZ274406, UAE84-benA: PZ290535, UAE84-CaM: PZ290536).

## 5. Discussion

These cases illustrate the diagnostic and clinical uncertainty associated with recovery of uncommon, initially non-sporulating molds from traumatic wounds. Although both patients sustained severe MVC-related polytrauma providing a plausible setting for environmental fungal inoculation, neither had sufficient evidence to establish invasive fungal disease. The cases therefore highlight two distinct challenges: achieving accurate species-level identification of unusual molds and determining the clinical significance of their recovery.

Post-traumatic IFI is an uncommon but severe complication of major trauma, characterized by progressive tissue necrosis, vascular invasion, and thrombosis [1,2,3,28]. MVC-associated cases have been reported particularly in the setting of extensive soft tissue injury, open fractures, tissue devitalization, and environmental contamination. In two series, MVCs accounted for 6 of 12 severe post-traumatic fungal infections and 6 of 12 cases of cutaneous mucormycosis, respectively [2,29,30,31]. Importantly, post-traumatic IFI may involve diverse molds beyond Mucorales, including *Aspergillus*, *Scedosporium*, *Curvularia*, and *Fusarium* [1,2,3,28,29,30,31].

Our patients shared several predisposing features with reported trauma-associated invasive fungal infections (IFI), including MVC-related polytrauma, extensive soft tissue disruption and devitalization, vascular injury, and repeated surgical intervention. Case 1 sustained a severe crush–degloving injury with wrist semi-amputation, extensive muscle destruction, and vascular compromise, whereas Case 2 sustained major upper-limb trauma requiring vascular reconstruction, fasciotomies, debridement, and skin grafting.

Despite these similarities, their subsequent courses differed from those typically described in established post-traumatic IFI. Neither patient developed progressive wound necrosis or other local or systemic features attributable to invasive fungal disease (IFD), although histopathologic assessment for tissue or vascular invasion was not performed. In Case 1, the mold was recovered only once, and the wound improved with surgical and antibacterial management without documented antifungal therapy. In Case 2, voriconazole was initiated following notification of the fungal culture result but discontinued after 15 days when the isolate was considered unlikely to represent true wound infection. Thus, although both cases occurred in a clinical setting associated with increased risk of post-traumatic IFI, evidence supporting invasive disease was lacking.

*S. asteroides* has been recovered from arid soils in Saudi Arabia and Egypt [17,32], supporting an ecology compatible with survival in the regional environment and the biological plausibility of traumatic inoculation. Although previously reported clinical cases have predominantly involved ocular disease [14,15], the severe crush–degloving injury in Case 1 provided a potential portal for environmental inoculation. However, no specific environmental exposure was documented, and neither the route of acquisition nor the clinical significance of the isolate could be established.

Identification of *S. asteroides* is complicated by the limited discriminatory value of conventional morphology. Historically, non-sporulating *Chaetomium*-like isolates have been reported as “mycelia sterilia” or provisionally assigned to morphologic groups such as *Papulaspora* because of the absence of diagnostic reproductive structures [14,16,19]. Bulbil- or papulospore-like cellular aggregates are non-specific and polyphyletic and may occur among phylogenetically unrelated fungi, further limiting morphology-based identification [16]. This diagnostic limitation was reflected in our Case 1 isolate, which was initially non-sporulating and subsequently developed limited, non-diagnostic structures, including heterogeneous hyphal morphology, papulospore-like elements, bulbil-like aggregates, and sparse phialidic conidiogenesis, none of which permitted reliable species-level identification. Molecular phylogenetic approaches have therefore been important in resolving such morphologically ambiguous isolates and establishing *Subramaniula* as a distinct lineage within the Chaetomiaceae [14,16]. Although more than 300 *Chaetomium*-like species have historically been described, modern molecular characterization is unavailable for many taxa, and the absence of living type or reference cultures for some historically described species further complicates taxonomic comparison [14,33]. Consistent with these limitations, MALDI-TOF MS did not provide a definitive identification of our isolate, whereas concordant ITS and D1/D2 sequence analysis, together with phylogenetic placement of UAE40 with the *S. asteroides* ex-type strain, supported species-level identification as *S. asteroides*.

The present case expands the clinical context in which *S. asteroides* has been recovered beyond the predominantly ocular presentations previously reported [14,15]. However, in the absence of histopathologic evidence of invasion and given wound improvement without documented antifungal therapy, its recovery should be regarded as being of uncertain clinical significance rather than evidence of established fungal infection. Reported clinical occurrences and available molecular identification data are summarized in Table 1.

*A. quadrilineatus* is a soil-dwelling thermotolerant fungus within section *Nidulantes*. The genus A comprehensive taxonomic revision recognized 446 accepted *Aspergillus* species across 6 subgenera, 27 sections, and 75 series, including 74 species within section *Nidulantes* [34]. Members of section *Nidulantes* are increasingly recognized as opportunistic pathogens, particularly in immunocompromised hosts, and some clinically relevant species exhibit reduced susceptibility to antifungal agents, including elevated amphotericin B [21,35,36,37]. *A. quadrilineatus* remains infrequently reported but has documented environmental and clinical occurrence across diverse geographic regions, including the Middle East. Molecular surveillance in Qatar has identified *A. quadrilineatus* among rare clinical *Aspergillus* isolates, with additional reports from the broader Middle Eastern region [5,9,13]. The present UAE isolate therefore adds to the limited regional documentation of *A. quadrilineatus* within the Arabian Gulf and highlights the contribution of multilocus molecular identification to recognizing uncommon *Aspergillus* species.

Species-level identification within *Aspergillus* section *Nidulantes* is complicated by close phylogenetic relatedness among cryptic taxa. Although ITS sequencing can support placement within section *Nidulantes*, it may lack sufficient discriminatory power for species-level identification, while *benA* may not reliably distinguish *A. quadrilineatus* from closely related species such as *A. sublatus* [8,21,38,39]. Multilocus sequence analysis incorporating more discriminatory loci, particularly *CaM* and *benA* alongside ITS, can therefore improve species-level resolution within this taxonomically complex group [8,21,38,39]. This limitation was directly reflected in our Case 2 isolate. ITS and *benA* showed high sequence similarity among *A. quadrilineatus* and closely related taxa, including *A. sublatus* and *A. nidulans*, but did not provide definitive species-level discrimination. In contrast, *CaM* provided greater discrimination among these related taxa, and concatenated analysis of ITS, *benA*, and *CaM* placed UAE84 within the *A. quadrilineatus* clade and distinguished it from closely related taxa within section *Nidulantes* (Figure 5). These findings demonstrate the value of integrating multiple loci rather than relying on a single marker for species-level identification of closely related species within section *Nidulantes*.

Antifungal susceptibility within section *Nidulantes* may vary among species, providing an additional rationale for species-level identification. Reduced susceptibility to amphotericin B has been reported among clinically relevant members of the section, and amphotericin B MIC_50_ and MIC_90_ values of 2 and 4 µg/mL, respectively, have been reported for *A. quadrilineatus* [35,36,37]. Although azoles remain recommended first-line agents for invasive aspergillosis [40], species-level identification may provide clinically relevant susceptibility information when uncommon *Nidulantes* isolates are recovered.

In contrast to previously reported disease-associated *A. quadrilineatus* infections [10,11,12], its recovery from the traumatic wound in Case 2 was not accompanied by clinical evidence establishing invasive fungal disease. Voriconazole was initiated following communication of the species-level identification but discontinued after 15 days when the isolate was considered unlikely to represent true wound infection. Thus, although multilocus molecular and phylogenetic characterization established the identity of the isolate, its etiologic role in the wound could not be established.

## 6. Limitations

Several limitations should be acknowledged. The retrospective nature of these cases and incomplete clinical documentation limited assessment of the significance of the recovered isolates. Most importantly, histopathologic assessment for fungal tissue or vascular invasion was not performed in either case, precluding definitive confirmation or exclusion of invasive fungal disease. In addition, microbiological evidence was limited to a single fungal recovery in each case, without serial cultures to demonstrate persistence. Longer-term clinical follow-up was also limited, particularly in Case 1. These limitations should be considered when interpreting the relationship between fungal recovery and the underlying traumatic wounds.

## 7. Conclusions

These cases demonstrate the value of integrated phenotypic and molecular approaches for resolving uncommon, initially non-sporulating molds recovered from traumatic wounds. Species-level identification of *S. asteroides* and *A. quadrilineatus* was achieved despite the limitations of conventional morphology and routine MALDI-TOF MS. Importantly, accurate species-level identification does not itself establish pathogenicity. Determining the significance of uncommon fungal isolates requires integration of microbiological findings with the clinical course and, where available, histopathologic evidence of tissue invasion.

## Figures and Tables

**Table 1:** Reported clinical isolation and identification of *Subramaniula asteroides*: a global summary of clinical and molecular findings.

Country	Clinical Presentation	Patient Details/ Risk Factor	Genes Used in Molecular Identification	Treatment	Outcome	Reference
USA	Keratitis	39 F; corneal trauma (wire injury), contact lens use	ITS, β-tubulin	Topical natamycin + fluconazole ± oral fluconazole	Clinical resolution; residual corneal scar	Vinod Mootha et al. [20]
India	Keratitis	Ocular trauma (plant material)	ITS, β-tubulin	Amphotericin B + fluconazole	Not reported	Ahmed et al. [14]
USA	Corneal ulcer	Not reported	ITS, β-tubulin	Not reported	Not reported	Ahmed et al. [14]
Spain	Keratitis (corneal exudate)	27 M	ITS	Not reported	Not reported	Ahmed et al. [14]
Spain	Endophthalmitis	47; immunosuppressed	ITS	Amphotericin B + fluconazole	Eye loss (evisceration)	Ahmed et al. [14]
Saudi Arabia	Skin infection	Not reported	ITS	Not reported	Not reported	Ahmed et al. [14]
Italy (infection acquired in Cape Verde)	Keratitis	65 M; no major comorbidity	β-tubulin	Voriconazole → Isavuconazole	Clinical resolution; vision improved	Cultrera et al. [15]
UAE (present case—UAE40)	Recovery from a post-traumatic left wrist wound following severe crush–degloving injury after MVC	55 M; stage 5 CKD; severe crush–degloving injury with extensive soft tissue disruption and vascular injury; multiple surgical interventions	ITS; D1/D2 domains of the 28S rRNA gene	Surgical management and antibacterial therapy; no antifungal therapy documented	Wound documented as clean and healing and subsequently clean and dry; outpatient wound follow-up through Day 35 with no clinical concerns documented; clinical significance of fungal recovery uncertain	Present study

## Data Availability

All ITS sequences generated in this study have been deposited in the NCBI GenBank database with accession numbers UAE40-ITS: PZ274405, UAE-40-D1-D2: PZ274423, UAE84-ITS: PZ274406, UAE-84-BT: PZ290535, and UAE-84-CAM: PZ290536.The data that support the findings of this study are available on request from the corresponding author.
